# Cost-Effectiveness of Telemonitoring and Self-Monitoring of Blood Pressure for Antihypertensive Titration in Primary Care (TASMINH4)

**DOI:** 10.1161/HYPERTENSIONAHA.118.12415

**Published:** 2019-04-15

**Authors:** Mark Monahan, Sue Jowett, Alecia Nickless, Marloes Franssen, Sabrina Grant, Sheila Greenfield, F. D. Richard Hobbs, James Hodgkinson, Jonathan Mant, Richard J. McManus

**Affiliations:** 1 From the Institute of Applied Health Research, University of Birmingham, United Kingdom (M.M., S.J., S. Greenfield, J.H.); 2 Nuffield Department of Primary Care Health Sciences, University of Oxford, United Kingdom (A.N., M.F., F.D.R.H., R.J.M.); 3 Translational Health Sciences, University of Bristol, United Kingdom (S. Grant); 4 Department of Public Health and Primary Care, University of Cambridge, United Kingdom (J.M.).

**Keywords:** blood pressure, cost-benefit analysis, probability, self-management

## Abstract

The use of self-monitoring of blood pressure, with or without telemonitoring, to guide therapy decisions by physicians for patients with hypertension has been recently demonstrated to reduce blood pressure compared with using clinic monitoring (usual care). However, both the cost-effectiveness of these strategies compared with usual care, and whether the additional benefit of telemonitoring compared with self-monitoring alone could be considered value for money, are unknown. This study assessed the cost-effectiveness of physician titration of antihypertensive medication using self-monitored blood pressure, with or without telemonitoring, to make hypertension treatment decisions in primary care compared with usual care. A Markov patient-level simulation model was developed taking a UK Health Service/Personal Social Services perspective. The model adopted a lifetime time horizon with 6-month time cycles. At a willingness to pay of £20 000 per quality-adjusted life year, self-monitoring plus telemonitoring was the most cost-effective strategy (£17 424 per quality-adjusted life year gained) compared with usual care or self-monitoring alone (posting the results to the physician). However, deterministic sensitivity analysis showed that self-monitoring alone became the most cost-effective option when changing key assumptions around long-term effectiveness and time horizon. Overall, probabilistic sensitivity analysis suggested that self-monitoring regardless of transmission modality was likely to be cost-effective compared with usual care (89% probability of cost-effectiveness at £20 000/quality-adjusted life year), with high uncertainty as to whether telemonitoring or self-monitoring alone was the most cost-effective option. Self-monitoring in clinical practice is cost-effective and likely to lead to reduced cardiovascular mortality and morbidity.

Hypertension is the foremost risk factor for cardiovascular disease and a significant contributor to mortality and morbidity worldwide.^1^ Blood pressure (BP) reduction using antihypertensive drugs reduces cardiovascular disease risk for hypertensive patients but many patients do not achieve optimal BP control.^2,3^ Self-management of treated hypertension, where the patient self-monitors their BP and titrates their own antihypertensive medication, has been shown to reduce BP and be cost-effective compared with routine care.^4,5^ However, until recently, the evidence for the use of self-monitoring to titrate antihypertensive medication by physicians was equivocal.^6,7^ The telemonitoring and/or self-monitoring of blood pressure in hypertension (TASMINH4) trial provided new evidence that physician titration using patient self-monitoring led to lower BP and that including telemonitoring led to lower BP quicker than self-monitoring alone.^8^ However, it remains unclear whether self-monitoring is cost-effective and whether the additional benefits from telemonitoring are sufficient to justify its use over and above self-monitoring alone on cost-effectiveness grounds.

The aim of this study was to estimate the long-term cost-effectiveness of general practitioner (GP) titration of antihypertensive medication using self-monitored BP to make hypertension treatment decisions in primary care, with or without telemonitoring, compared with usual care, from a National Health Service (NHS)/Personal Social Services perspective.

## Methods

Requests for the data in this article should be addressed to the corresponding author.

Full details of the TASMINH4 trial have been published elsewhere.^8,9^ Briefly, this was a randomized controlled trial assessing the efficacy of using self-monitored BP, with or without telemonitoring, to guide antihypertensive titration in primary care. Eligible patients were hypertensive, aged >35 years, with a clinic BP >140/90 mm Hg and willing to self-monitor their BP. Across 138 GP practices, 1182 patients were randomized (1:1:1) to antihypertensive titration using self-monitoring, telemonitoring, or usual care (clinic BP).

The self-monitoring and telemonitoring interventions are described in detail in the section on model comparators. The primary outcome for the trial was clinic systolic BP measured by a research nurse at 12 months. Cost and resource utilization data were collected during the trial through notes review.^9^ It was not possible to capture primary care workload not recorded in the clinical records.

A Markov patient-level simulation was undertaken in TreeAge 2018 (TreeAge Software, Inc, Williamstown, MA) to model the different strategies. This type of Markov model tracks the costs and consequences of individual patients passing through the model, with characteristics (taken from TASMINH4 patient-level data) free to vary between patients. The model was run over a lifelong (maximum of 65 years; minimum trial inclusion criteria was age 35) time horizon to capture all relevant long-term costs and consequences.

### Study Population

Each patient had characteristics created by randomly sampling the trial patient-level data by means of a uniform distribution. These characteristics affected their probability of subsequent model events (eg, males had a higher cardiovascular disease risk relative to females). The model was run with a large number of simulated patients (50 000) to account for interpatient variability and to adequately model a representative clinical population.

### Model Comparators and Costs

After randomization, trial participants booked a primary care appointment for a BP check and a medication review. GPs were free to make management decisions and drug choice in all 3 randomized groups. For usual care, GP antihypertensive treatment decisions were based on clinic BP readings. All 3 strategies included the cost of baseline medication review, subsequent primary care consultations and antihypertensive prescriptions (Table 2).

**Table 1.**
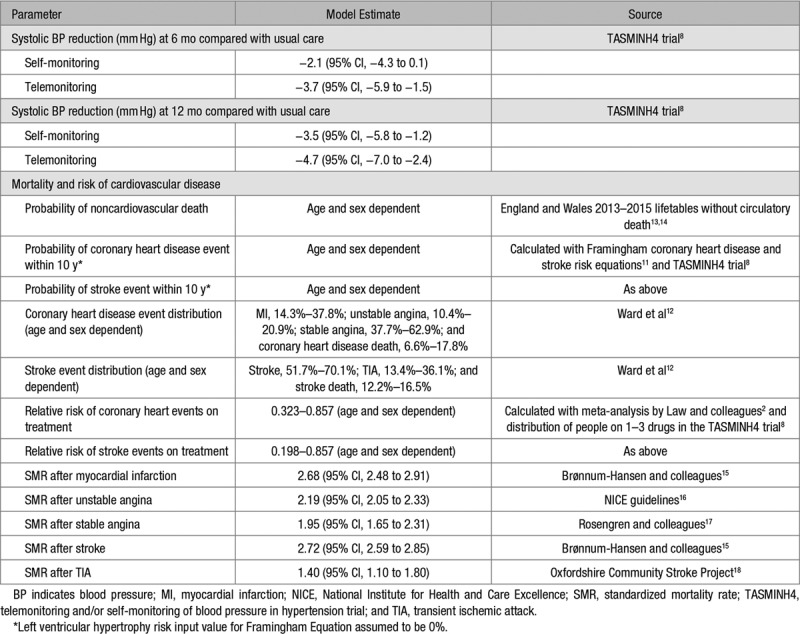
Model Parameters

**Table 2.**
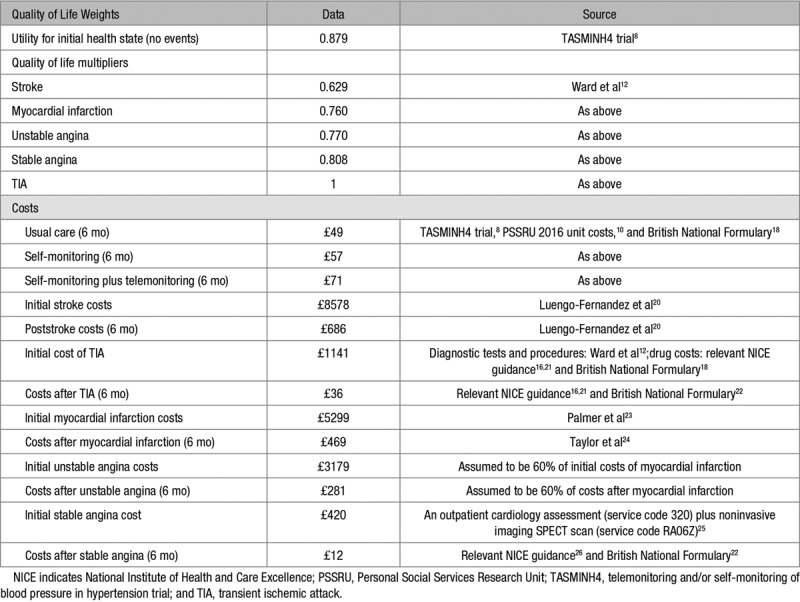
Quality of Life and Costs

Patients in the self-monitoring alone group were trained to self-monitor their own BP by a nurse and asked to post the readings to their practice every month. Additional costs included 15 minutes self-monitoring training, the BP monitor and 2-part copy forms with reply paid envelopes (Table 2). Participants in the telemonitoring group were trained, again by a nurse, to self-monitor their own BP and to use an SMS text-based telemonitoring service, with web-based data entry back-up to send the BP readings. Additional costs included for this strategy were 15 minutes self-monitoring training, 25 minutes telemonitoring training, the BP monitor, and telemonitoring server costs (Table 2). The telemonitoring system included reminders, warnings in the case of very high or very low readings, and a secure website that GPs could use to review their patients’ measurements.^8^ For the self-monitoring and telemonitoring groups, the GP was asked to use the self-monitored BP readings to guide any antihypertensive treatment decisions.

Costs of modeled cardiovascular events (detailed in the Model Structure section), including initial acute care costs and long-term care, were obtained from previously published work and standard reference costs (Table 2). Costs are reported in 2015/2016 prices (the trial took place 2014–2016) and inflated where applicable using the Hospital and Community Health Services Index.^10^ Training and equipment, annuitized over 5 years, were included in the self-monitoring and telemonitoring costs.

### Model Structure

**Figure 1.**
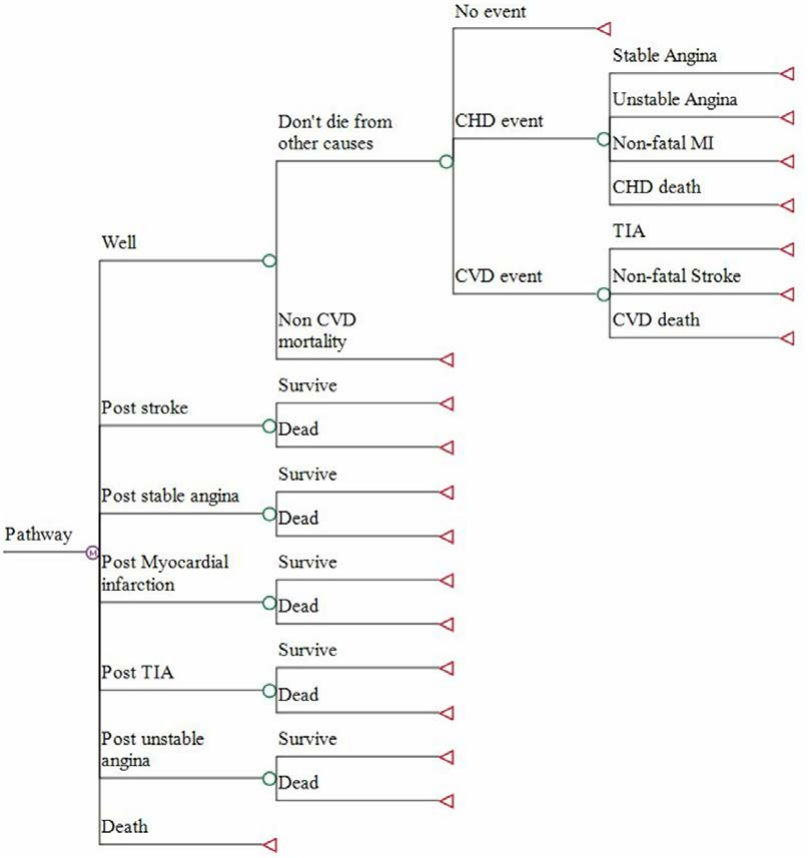
Model patient pathways. CHD indicates coronary heart disease; CVD, cardiovascular disease; MI, myocardial infarction; and TIA, transient ischemic attack.

The patient pathway is shown in Figure 1. All patients started in the well/no event health state. Within a 6-month time cycle, a patient had a risk of suffering a fatal or nonfatal cardiovascular event or dying from other causes. The possible cardiovascular events in the model were stable angina, unstable angina, stroke, myocardial infarction, and transient ischemic attack. Ten-year cardiovascular risk was calculated for each individual patient, with the distribution of coronary heart disease and stroke events dependent on age and sex.^11,12^ Patients who suffered a nonfatal cardiovascular event transitioned to a postevent cardiovascular health state and additional clinical events were not modeled. Once a cardiovascular event had occurred, mortality risk was adjusted accordingly. The impact of each intervention in terms of event reduction was applied as a relative risk, taking into account the mean differences in systolic BP observed in the TASMINH4 trial. Compared with usual care, the mean differences (at 6 and 12 months, respectively) were −2.1 and −3.5 mm Hg for self-monitoring alone and −3.7 and −4.7 mm Hg for telemonitoring.^2^ Individual relative risks were applied, taking into account age, sex, and baseline clinic systolic BP. In the base-case analysis, it was assumed that the 12-month differences were maintained over the patient lifetime. Model inputs are detailed in Table 1.

Half-cycle correction was applied to model costs and outcomes. Future costs and outcomes were discounted at an annual rate of 3.5% as recommended by the National Institute of Health and Care Excellence.^19^

### Model Outcomes

Health-related quality of life outcomes were modeled in quality-adjusted life years (QALYs), taking into account both quality of life and survival. Utility scores for health states are detailed in Table 2. Each patient’s initial quality of life was the overall mean EQ-5D-5L^27^ score at baseline taken from the TASMINH4 trial,^8^ calculated using UK tariff values.^28^ Utility values for cardiovascular events were applied multiplicatively to baseline utility scores. Sex-specific life tables were used to determine the probability of death at different ages, with adjustment to avoid double counting of circulatory deaths.^13,14^

### Analysis

A cost-utility analysis from an NHS/Personal Social Services perspective was undertaken to estimate Incremental Cost-Effectiveness Ratios (ICERs). An ICER was calculated as the difference in costs divided by the difference in QALYs of 2 strategies, with results presented as cost per QALY gained. The cost-effectiveness of an intervention was considered in relation to the lower National Institute of Health and Care Excellence threshold of £20 000 per QALY gained.^29^ In line with convention, strategies were ordered from least to greatest cost, with each strategy compared against the next more costly strategy, and strategies which were dominated or extendedly dominated subsequently excluded.^30^ Probabilistic Sensitivity Analysis (PSA) was undertaken to assess parameter uncertainty.^31^ Where possible, distributions were attached to probabilities, utilities, and costs in the model. Beta distributions were attached to probabilities and utilities, and gamma distributions were attached to costs. Log-normal distributions were used for the relative risks associated with BP reduction from the interventions and relative risks for mortality. The model was run for 10 000 iterations across 1000 patients, and the results are expressed as a Cost-Effectiveness Acceptability Curve.^32^ The Cost-Effectiveness Acceptability Curve shows graphically the probability of cost-effectiveness for all strategies across a range of cost per QALY thresholds.

### Deterministic Sensitivity Analyses

The impact of changing model assumptions was undertaken using deterministic sensitivity analysis to assess model robustness.^31^ The following scenarios were explored:

1. The time horizon was varied from a lifetime time horizon to between 5 and 20 years following the end of the trial.
2. The duration of the impact of BP reduction because of self-monitoring alone and self-monitoring plus telemonitoring was varied from the base-case assumption of lifetime impact.

Post hoc, a further 2 sensitivity analyses were undertaken given uncertainty shown by the PSA as to which option was most cost-effective. These analyses were designed to investigate the effect of bringing the cost of the 2 interventions closer to assess the influence of this on ICER given that:

1. Additional administrative work not captured in the clinical record was likely to have been an issue for self-monitoring alone from our linked qualitative work (Sabrina Grant, personal communication, 2018). This is because someone had to deal with the paper self-monitoring records, add the data onto the clinical record system, and work out an average BP. As the exact time taken for this task was unknown, alternative scenarios for self-monitoring were assessed by including the assumed cost of a GP receptionist’s time for data entry in the self-monitoring group using 2 different time durations (5 or 10 minutes per month per patient receptionist time).
2. A national rollout of telemonitoring was considered likely to lead to reductions in the cost of telemonitoring from economies of scale compared with the relatively small scale used in the trial. Alternative cost scenarios for telemonitoring were, therefore, evaluated by reducing the annual cost of telemonitoring by £10 and £20 per year respectively.

Making the telemonitoring more expensive or the self-monitoring less expensive were not considered likely enough scenarios to be modeled. Both would have tended to increase the difference between the 2 interventions in cost and, therefore, favored self-monitoring alone.

## Results

**Figure 2.**
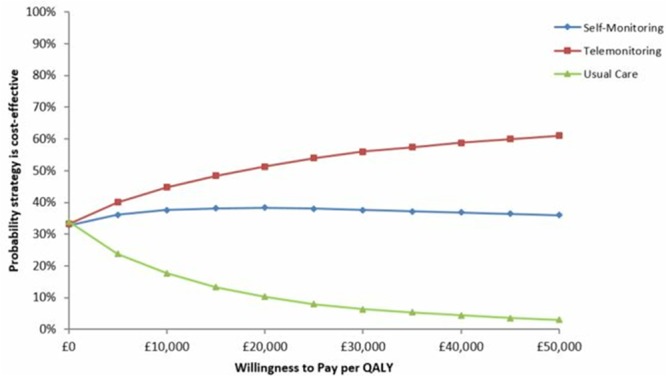
Cost-effectiveness acceptability curve of antihypertensive titration options. QALY indicates quality-adjusted life year.

Base-case findings are reported in Table 3, ordering strategies from least to greatest cost, with each strategy compared against the next more costly strategy. Self-monitoring alone was cost-effective compared with usual care, with an ICER of £3035 per QALY gained. Telemonitoring resulted in the most QALYs and was cost-effective at the £20 000/QALY threshold compared with self-monitoring alone, with an ICER of £17 424 per QALY. The results of the PSA are shown in Figure 2. At a willingness to pay of £20 000 per QALY, telemonitoring was the most cost-effective option in 51% of iterations while self-monitoring was the most cost-effective option in 38% of iterations. This shows that while self-monitoring (with or without telemonitoring) had a high probability of being cost-effective, there was high uncertainty as to which specific option (telemonitoring or not) was the most cost-effective compared with usual care.

**Table 3.**
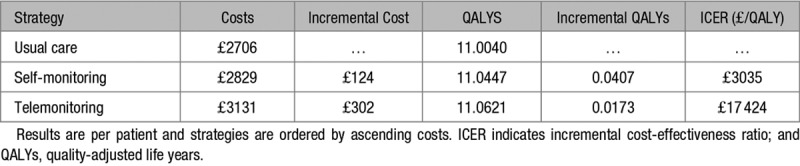
Base-Case Results

The results of deterministic sensitivity analysis are shown in Table 4. When the model time horizon was reduced to 20, 10, and 5 years from the end of the trial respectively, the self-monitoring strategy remained cost-effective compared with usual care. The results were also robust when reducing the effect of the intervention from lifetime to 10, 5, and 3 years, respectively. However, when the effect was assumed to be only an additional 2 years, the self-monitoring strategy was no longer cost-effective compared with usual care. For all these scenarios, telemonitoring was no longer cost-effective compared with self-monitoring alone.

**Table 4.**
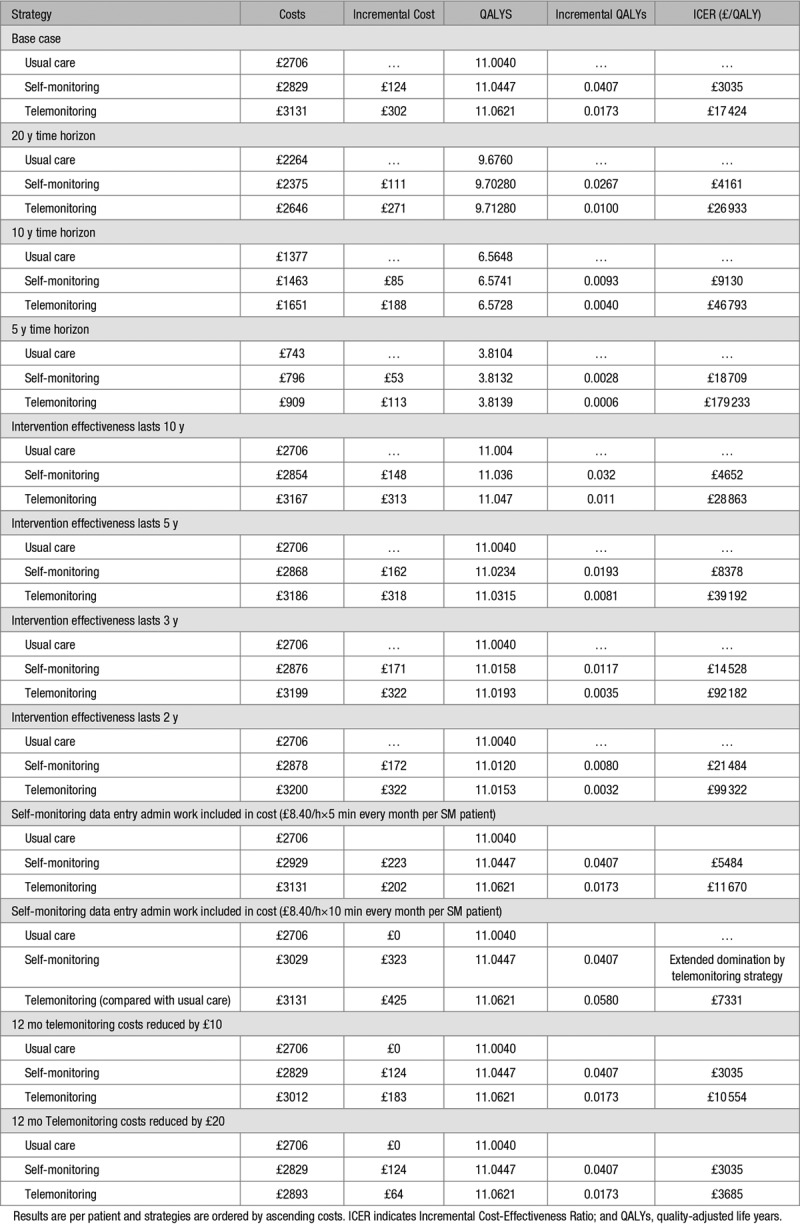
Sensitivity Analysis Scenarios

If an additional 5 minutes of administration time per patient per month for managing paper results were added to self-monitoring, when compared with the base-case results, this resulted in a higher ICER for self-monitoring versus usual care (£5484/QALY) and a lower ICER for telemonitoring (£11 670/QALY) versus self-monitoring. When this additional time was increased to 10 minutes per month, telemonitoring demonstrated extended dominance over self-monitoring alone and had an ICER of £7331/QALY compared with usual care. If the cost of telemonitoring was reduced annually by £10 per patient, self-monitoring remained cost-effective at an ICER of £3305/QALY and the ICER for telemonitoring versus self-monitoring reduced to £10 554/QALY compared with the base-case result. A decrease in the annual cost of telemonitoring by £20 per patient resulted in a much lower ICER of £3685/QALY when compared with self-monitoring.

## Discussion

### Main Findings

This model-based economic evaluation has demonstrated that antihypertensive titration by physicians in primary care using patient self-monitoring of BP data is cost-effective compared with usual care, provided that the BP improvements associated with self-monitoring observed here last at least 3 years after the end of the trial (which lasted 1 year). With longtime horizons and the assumption of ongoing efficacy, telemonitoring was cost-effective over and above self-monitoring alone at the lower National Institute of Health and Care Excellence threshold of £20 000 per QALY. However, this was not the case with shorter durations of action or shorter time horizons, although the results were finally balanced and sensitive to small increases in costs for self-monitoring (5–10 minutes extra work per month per person) or small reductions in costs for telemonitoring (£10–20 per patient per year), both of which seem plausible. PSA reflected these issues and indicated high levels of uncertainty as to whether self-monitoring alone or in addition to telemonitoring was the most cost-effective.

### Strengths and Weaknesses

The model was based on the results of TASMINH4, the only primary care trial of physician antihypertensive titration utilizing self-monitoring and/or telemonitoring BP measurements, with currently recommended targets for self-monitored BP.^33^ Furthermore, patients in the study were drawn from a wide range of primary care practices in varied settings across England, and therefore, the results are likely to be generalizable, at least in a UK NHS context. Patients in the trial were recruited on the basis of standardized clinic BP measurements which might have reduced the white coat effect; however, this would tend to suggest that self-monitoring might be even more cost-effective in a population chosen on the basis of normal clinic measurement where the prevalence of white coat hypertension might be higher. Furthermore, although alternative model parameter estimates would be required to produce precise country-specific cost-effectiveness estimates, systematic reviews suggest similar effect sizes in self-monitoring trials with cointerventions in the UK and internationally.^34^ Therefore, given that self-monitoring reduced health service costs, and these are generally lower in the NHS than in other comparable developed countries, these results retain wide relevance.

The economic analysis used a patient-level Markov simulation^35^ in assessing the costs and consequences of the interventions. This is preferable to the more common Markov cohort model, which is limited by the use of a population with a homogenous set of characteristics, because of the model’s inherent lack of memory.^35^ The patient-level simulation was able to draw on individual patients’ trial data, with characteristics allowed to vary (using tracker variables to overcome the lack of Markov memory issues), and therefore, made the findings representative of the TASMINH4 trial population. The modeling method was also more efficient by avoiding the construction of a large number of Markov health states.

The base-case model assumed extrapolation of the effectiveness of the interventions beyond the 12 months of the trial to a lifetime effect. However, sensitivity analyses demonstrated that as long as the intervention effects persisted for an additional 3 years, self-monitoring would still be cost-effective. This seems a reasonable assumption, as the BP differences between the 2 intervention groups and the usual care group widened between 6 months and 1 year in the TASMINH4 trial^8^ and similarly in other self-monitoring trials.^36,37^ Undertaking the analysis from an NHS/Personal Social Services perspective, in line with the National Institute of Health and Care Excellence reference case, may mean that broader health service and societal benefits of telemonitoring have been neglected.^19^ In the future, telemonitoring has the potential to be integrated into electronic health care records, allowing seamless management by primary care nurses and GPs, and also facilitating remote access to a doctor. Furthermore, self-monitoring may be more beneficial to individuals in both working age populations and those requiring a carer, reducing, for example, time off work to attend appointments either for oneself or a relative. Conversely, in the current analysis, any additional time required by GPs to process the results from self-monitoring or telemonitoring was not captured. The sensitivity analyses showed that inclusion of unrecorded costs of processing manual self-monitoring records, or economies of scale resulting in cheaper telemonitoring, would make telemonitoring more cost-effective. Results of the PSA illustrated the considerable uncertainty in the results with similar probabilities of cost-effectiveness for both interventions, in part, driven by the uncertainty in the estimates of BP lowering. Finally, the risk of further events once someone had an initial cardiovascular event was not modeled, and baseline cardiovascular disease (affecting around 5% of participants) was not included in the model, hence potential additional benefits of secondary preventative treatment, which might be expected to favor self-monitoring, were ignored.

### Findings in the Context of Existing Literature

A previous United Kingdom-based economic evaluation undertook a cost-effectiveness analysis of a trial of 6 months of telemonitoring versus usual care.^38^ However, it was a within-trial analysis reporting results in natural units (cost per 1 mm Hg systolic BP point reduced) and did not undertake any longer-term modeling of costs and consequences (including QALYs) arising from the intervention. Direct comparison of within-trial results at 6 months shows that compared with their published ICER of £25.60/mm Hg, both self-monitoring and telemonitoring in TASMINH4 were much more cost-effective compared with usual care (£3.81/mm Hg and £5.95/mm Hg, respectively).

Maciejewski et al^37^ conducted a follow-up analysis of their 18-month long United States-based trial which included telemedicine and home BP monitoring to assess longer-term clinical and economic outcomes. Results showed that the BP improvements in the trial were maintained for at least a further 18 months after trial completion (because of worsening control in the usual care arm), but that health care costs did not decrease. Similar results have been recently published from a self-monitoring/community pharmacist intervention.^36^ Therefore, the extrapolation of the TASMINH4 results over a numbers of years seems to be reasonable.

A previous trial by our group (TASMINH2)^5^ in a similar patient population found that self-management with telemonitoring and self-titration was cost-effective compared with usual care (ICER £1624 per QALY for men and £4923 for women), which is in accordance with the current findings that telemonitoring was cost-effective when compared to usual care.

### Implications for Clinical Practice

The TASMINH4 trial demonstrated that self-monitoring, with or without telemonitoring, led to significantly lower BP compared with usual care.^8^ The case for self-monitoring of hypertension (with or without telemonitoring) has been further supported by this cost-effectiveness analysis but, ultimately, the decision whether or not to implement telemonitoring in primary care for the management of hypertension may need to include factors such as practice logistics and patient preferences not included in this modeling exercise, as well as a careful focus on procurement prices.

### Perspectives

Antihypertensive titration by physicians in primary care using self-monitoring of BP, with or without telemonitoring, is cost-effective compared with usual care, provided self-monitoring has ongoing effects on BP reduction for at least 3 additional years. The decision as to whether or not to use telemonitoring is finely balanced. However, both digital and manual approaches to implementation of self-monitoring are cost-effective relative to usual care, so availability of both may be appropriate where patient choice determines which is used.

## Acknowledgments

We acknowledge the support of the Primary Care Clinical Trials Unit Oxford. Karen Biddle, Siobhan Milner, and Carla Betts provided additional administrative support. David Yeomans and Derek Shaw served as patient representatives for the trial management and steering groups. Dr Oliver Gibson developed a previous system for telemonitoring that was helpful in the development of the current system, and Dr Mauro Santos and Carmelo Valderes provided technical support in the latter part of the trial. Additional members of the trial steering group were Mike Moore (chair), Pip Logan, and Dr Chris Clark. Members of the data monitoring committee were Dr Martyn Lewis (chair), Dr Emma Bray, and Sarah Purdy.

## Sources of Funding

This work was funded by the National Institute for Health Research (NIHR) via a Programme Grant (RP-PG-1209–10051). R.J. McManus held an NIHR Professorship and leads the self-management theme of the NIHR Oxford Collaboration for Leadership in Applied Research in Health and Care (CLAHRC). F.D.R. Hobbs acknowledges part support from the NIHR School for Primary Care Research (SPCR), the NIHR Oxford CLARHC, and the NIHR Biomedical Research Centre (BRC), Oxford. The views and opinions expressed are those of the authors and do not necessarily reflect those of the NHS, NIHR, or the Department of Health and Social Care.

## Disclosures

R.J. McManus received blood pressure monitors from Omron which were used in the TASMINH4 trial. The other authors report no conflicts.

## Novelty and Significance

### What Is New?

- Self-monitoring of blood pressure is increasingly utilized in both primary care and specialized clinic settings, but no previous study has assessed cost-effectiveness.
- This study used a modeling approach to consider the long-term cost-effectiveness of using self-monitoring of blood pressure (with or without telemonitoring) to guide treatment decisions by primary care physicians for patients with hypertension.

### What Is Relevant?

- Using self-monitoring of blood pressure to guide treatment decisions is cost-effective compared with usual care, however, there is uncertainty whether self-monitoring is most cost-effective when used alone or when combined with telemonitoring.

### Summary

Economic modeling suggests that antihypertensive titration by physicians in primary care using self-monitoring of blood pressure (with or without telemonitoring) would be cost-effective compared with usual care.

Healthcare organizations/payees should be encouraged to implement self-monitoring of blood pressure into usual hypertension care.
